# dUTPase inhibition augments replication defects of 5-Fluorouracil

**DOI:** 10.18632/oncotarget.15785

**Published:** 2017-02-28

**Authors:** Anna Hagenkort, Cynthia B.J. Paulin, Matthieu Desroses, Antonio Sarno, Elisée Wiita, Oliver Mortusewicz, Tobias Koolmeister, Olga Loseva, Ann-Sofie Jemth, Ingrid Almlöf, Evert Homan, Thomas Lundbäck, Anna-Lena Gustavsson, Martin Scobie, Thomas Helleday

**Affiliations:** ^1^ Division of Translational Medicine and Chemical Biology, Science for Life Laboratory, Department of Medical Biochemistry and Biophysics, Karolinska Institutet, Stockholm, Sweden; ^2^ Department of Cancer Research and Molecular Medicine, Norwegian University of Science and Technology, Trondheim, Norway; ^3^ Chemical Biology Consortium Sweden, Division of Translational Medicine and Chemical Biology, Science for Life Laboratory, Department of Medical Biochemistry and Biophysics, Karolinska Institutet, Stockholm, Sweden

**Keywords:** dUTPase, 5-Fluorouracil, DNA replication, combination therapy

## Abstract

The antimetabolite 5-Fluorouracil (5-FU) is used in the treatment of various forms of cancer and has a complex mode of action. Despite 6 decades in clinical application the contribution of 5-FdUTP and dUTP [(5-F)dUTP] and 5-FUTP misincorporation into DNA and RNA respectively, for 5-FU-induced toxicity is still under debate.

This study investigates DNA replication defects induced by 5-FU treatment and how (5-F)dUTP accumulation contributes to this effect. We reveal that 5-FU treatment leads to extensive problems in DNA replication fork progression, causing accumulation of cells in S-phase, DNA damage and ultimately cell death. Interestingly, these effects can be reinforced by either depletion or inhibition of the deoxyuridine triphosphatase (dUTPase, also known as DUT), highlighting the importance of (5-F)dUTP accumulation for cytotoxicity.

With this study, we not only extend the current understanding of the mechanism of action of 5-FU, but also contribute to the characterization of dUTPase inhibitors. We demonstrate that pharmacological inhibition of dUTPase is a promising approach that may improve the efficacy of 5-FU treatment in the clinic.

## INTRODUCTION

Even after six decades, targeting thymidine synthesis is still one of the most successful strategies to treat cancer [1, 2]. Thymidylate synthase (TS) converts dUMP to dTMP, utilizing 5,10-methylenetetrahydrofolate (5,10-CH_2_THF) as methyl-donor. dTMP is the precursor for dTTP production, making this reaction essential for thymidine synthesis [3]. TS forms a homodimer, which contains both a substrate (dUMP), and a cofactor (5,10-CH_2_THF) binding pocket [4]. Inhibiting the function of TS can therefore be achieved by nucleobase- and nucleoside analogs (*e.g*. 5-Fluorouracil (5-FU) or FUdR), as well as antifolates (*e.g*. Pemetrexed) [3, 5].

The 5-fluoro-substituted uracil analogs are metabolized to 5-FdUMP, which binds and thereby occupies the TS-substrate pocket [4]. Inhibition of TS leads to depletion of thymidine but also accumulation of the substrate dUMP, which is phosphorylated to dUTP. In addition, conversion of 5-FU to 5-FdUTP further elevates uracil levels. The increased dUTP/dTTP and 5-FdUTP/dTTP ratios promote uracil misincorporation into DNA by DNA-polymerases [6]. Subsequent attempts of futile DNA repair eventually lead to cell death [6–10]. Besides DNA-associated toxicity, incorporation of the 5-FU metabolite 5-FUTP into RNA has been shown to contribute to cell death [11–14]. However, the metabolism and working mechanism of fluoropyrimidines are complex and the contribution of each of these components for toxicity is often debated.

Deoxyuridine triphosphatase (dUTPase, also known as DUT) circumvents high levels of uracil in the biosynthetic pool by hydrolyzing dUTP to dUMP and pyrophosphate. This reaction additionally supplies TS with its substrate dUMP [15]. Despite the selective binding pocket of dUTPase, the 5-FU metabolite 5-FdUTP has been shown to be a substrate for this enzyme [16]. The physiological function of dUTPase is to reduce dUTP accumulation and prevent misincorporation of the non-canonical nucleotide into DNA. However, from a treatment perspective, this activity could hamper the therapeutic success of 5-FU.

Several studies have shown that dUTPase levels significantly influence TS-based treatment response. Ectopical overexpression of *E. coli* dUTPase induced resistance to FUdR in human cells [17]. In contrast, depletion of dUTPase increased response to FUdR and Pemetrexed [18, 19]. dUTPase expression also inversely correlated with sensitivity to TS inhibitor ZD9331 [20]. Moreover, in patient samples, high nuclear dUTPase expression was associated with both resistance to 5-FU therapy [21] and metastasis [22]. Interestingly, a dUTPase inhibitor was reported to sensitize cancer cells to 5-FU treatment in a xenograft setting [23].

Despite adjusting treatment regimens and improving TS-based therapies, a large number of patients still exhibit intrinsic or acquired treatment resistance [2]. Further clarification of the 5-FU mechanism of action in combination with dUTPase inhibitors is required to improve the treatment outcome. Here, we demonstrate that 5-FU treatment induces DNA replication defects. Pharmacological inhibition and knockdown of dUTPase further augment 5-FU induced perturbations at the replication fork, DNA damage and cell death, highlighting the importance of 5-FdUTP and dUTP [(5-F)dUTP] and dUTPase for 5-FU-induced cytotoxicity.

## RESULTS

### dUTPase depletion increases cytotoxicity of 5-FU in SW620 colorectal cancer cells

**Figure 1:**
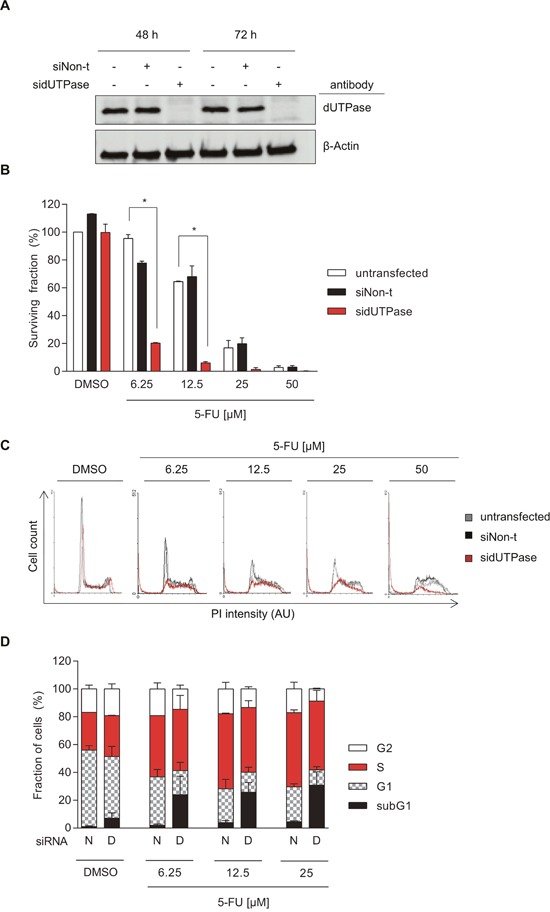
Depletion of dUTPase increases cytotoxicity of 5-FU in colorectal cancer cells **(A)** Representative Western Blot assessing dUTPase expression after 48 and 72 hours of siRNA treatment using dUTPase specific siRNA (sidUTPase) or a non-targeting siRNA control (siNon-t). β-Actin was used as a loading control. **(B)** Clonogenic survival of dUTPase depleted cells compared to siNon-t transfected or untransfected controls, treated for 48 hours with increasing concentrations of 5-FU. Data shown as average ± SEM from two independent experiments performed in triplicate. Statistical significance between untransfected and sidUTPase was determined by using a two-tailed t-test. **(C)** FACS analysis highlighting cell cycle alterations induced by 5-FU treatment in sidUTPase and siNon-t transfected cells. After 48 hours of siRNA transfection, cells were re-seeded and, 24 hours later, treated for 48 hours with increasing concentrations of 5-FU. DNA content was stained with PI and analyzed by FACS. Representative histograms are shown. Abbreviation: AU: arbitrary unit. **(D)** Quantification of the FACS experiment in Figure 1C. Data shown as average ± SEM from two independent experiments. Abbreviations: N: siNon-t, D: sidUTPase.

To understand the importance and consequences of (5-F)dUTP accumulation during 5-FU treatment we depleted dUTPase in SW620 colorectal cancer cells using siRNA-mediated knockdown. Transfection with a dUTPase specific siRNA (sidUTPase) depleted protein levels after 48 hours (Figure 1A and Supplementary Figure 7A). A non-targeting siRNA (siNon-t) control was compared to untransfected cells to rule out non-dUTPase related effects from the siRNA transfection.

dUTPase depleted and control cells were exposed to 5-FU for 48 hours and re-seeded to assess their ability to form colonies. Whereas dUTPase depletion by itself had no effect on cell survival, it significantly increased the cytotoxic effect of 5-FU, when compared to the untransfected or siNon-t transfected cells (Figure 1B).

To further understand the mechanism of toxicity, dUTPase-depleted and control cells were treated for 48 hours with 5-FU and the cell cycle was analyzed by FACS. While 5-FU treatment of up to 25 μM accumulated cells in S-phase, it had only minimal cytotoxic effects, indicated by a minor increase in the subG1 population (Figure 1C-1D). dUTPase depletion, upon 5-FU treatment, increased the subG1 population already at the lowest dose of 5-FU tested from 2 to 24% (6.25 μM of 5-FU). Notably, depletion of dUTPase by itself resulted in a small increase of subG1, S- and G2-phase cells and a reduction in the G1 population. No difference in the subG1 population was observed between the untransfected and siNon-t transfected cells (Supplementary Figure 1).

### dUTPase depletion increases 5-FU-induced S-phase arrest of the cell cycle

**Figure 2:**
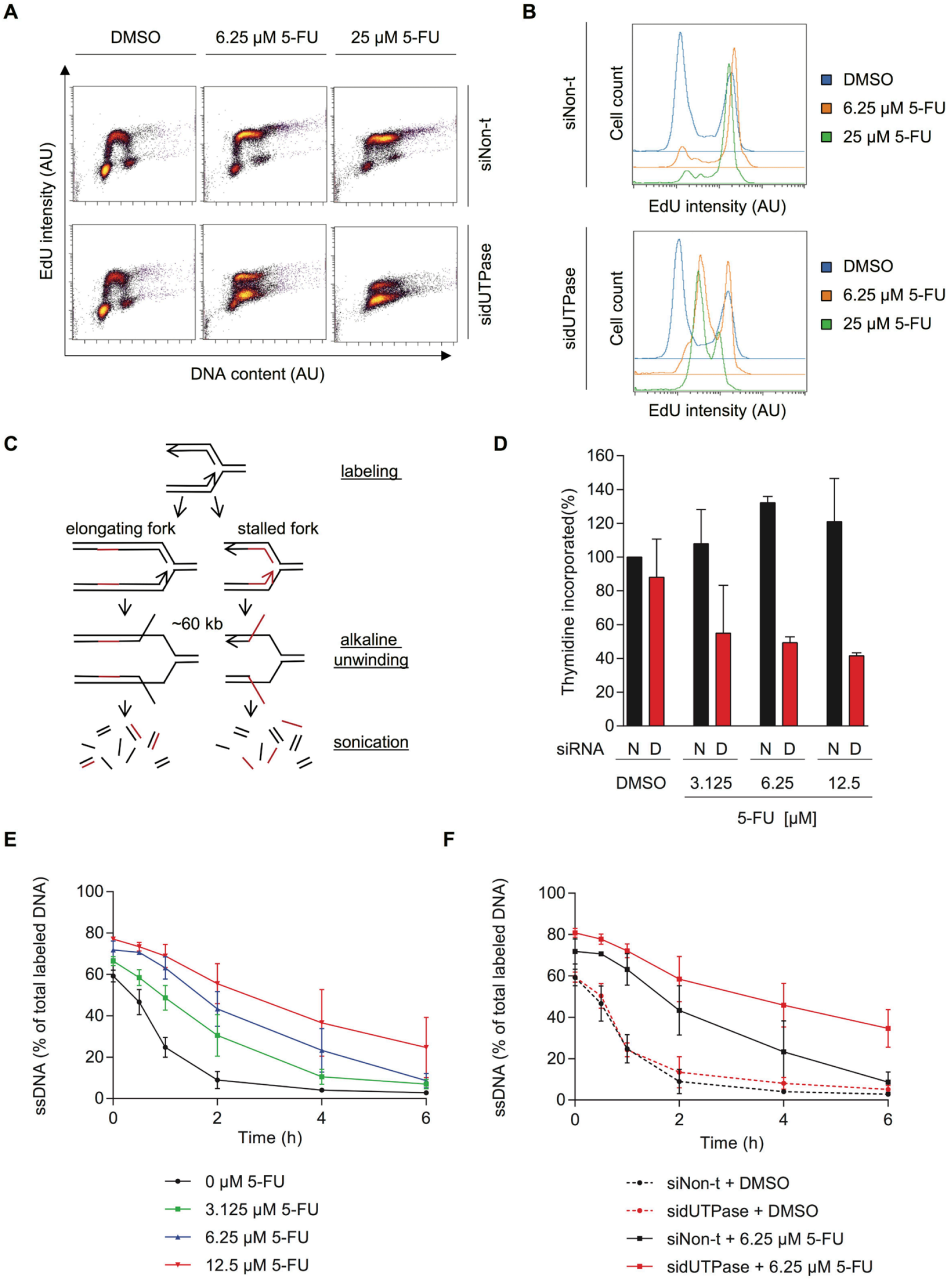
5-FU treatment accumulates cells in S-phase by decreasing replication fork progression, which can be accentuated by dUTPase depletion **(A)** FACS analysis of incorporated EdU after the indicated treatments. After 72 hours of siRNA transfection, cells were treated for 48 hours with the indicated concentration of 5-FU. Replication was labeled by addition of 1 μM EdU for 30 min, which was analyzed by FACS. EdU intensity is depicted on the y-axis and DNA content (To Pro intensity) on the x-axis. A representative experiment is shown. Abbreviation: AU: arbitrary unit. **(B)** Representative histograms displaying the EdU intensity over cell count (events) of the experiment shown in Figure 2A. Abbreviation: AU: arbitrary unit. **(C)** Schematic illustration of the ADU technique. **(D)** Total amount of ^3^H-thymidine incorporated within 30 min after 48 hours of 5-FU treatment in dUTPase depleted and control cells. Data shown as average ± SEM from two independent experiments. Data shown as average ± SEM from two independent experiments. **(E)** Replication fork progression, measured by the ADU technique, of SW620 cells treated with increasing concentrations of 5-FU for 48 hours. **(F)** Replication fork progression, measured by the ADU technique, in dUTPase depleted and control cells treated for 48 hours with 5-FU. For E-F: Percentage of radioactive label in the single stranded DNA (ssDNA) fraction compared to the total signal (ssDNA plus double stranded DNA) is depicted on the y-axis. Data shown as average ± SEM from two independent experiments.

To determine the number of S-phase cells in the cell cycle, we next measured EdU incorporation into DNA. As expected, 5-FU treatment alone increased the amount of cells in S-phase, as demonstrated by more incorporation of EdU into DNA (Figure 2A-2B). Interestingly, dUTPase depletion during the 5-FU treatment led to reduced amount of EdU being incorporated.

We further analyzed DNA replication using the alkaline DNA unwinding (ADU) technique (Figure 2C) [24]. In this assay, replicating forks are pulse labeled by incorporation of ^3^H thymidine, followed by a fresh media treatment. At increasing time points, DNA is unwound for about 60 kb by addition of an alkaline solution. The genome is subsequently fragmented into 3 kb pieces using ultrasonic treatment. This treatment creates a fraction of single stranded DNA (ssDNA) close to the replication fork and double stranded DNA (dsDNA) away from the fork. The radioactive label shifts from the ssDNA to the dsDNA fraction as the fork moves forward. The comparison of radioactivity in the ssDNA compared to the dsDNA fraction is therefore a measure of replication fork progression.

In line with the EdU data, 48 hours of 5-FU treatment led to increased incorporation of total radioactive thymidine at time zero compared to untreated cells, which can be explained by the increased amount of cells in S-phase (Figure 2D). Furthermore, dUTPase depletion reduced the total amount of thymidine incorporated, supporting the FACS analysis. Since ^3^H thymidine was used it would not require dUTPase activity to be introduced into DNA. Therefore, we conclude a true reduced fork rate following dUTPase treatment.

As time progressed and the replication fork proceeded, the radioactive signal moved from the ssDNA to the dsDNA fraction. When cells were treated with increasing concentrations of 5-FU, the ssDNA to dsDNA exchange was delayed in a dose-dependent manner, indicating reduced replication fork speed (Figure 2E). In dUTPase depleted cells, an even slower exchange was observed, indicating on average slower DNA replication, compared to the siNon-t control (Figure 2F).

### dUTPase depletion increases 5-FU-induced replication defects

**Figure 3:**
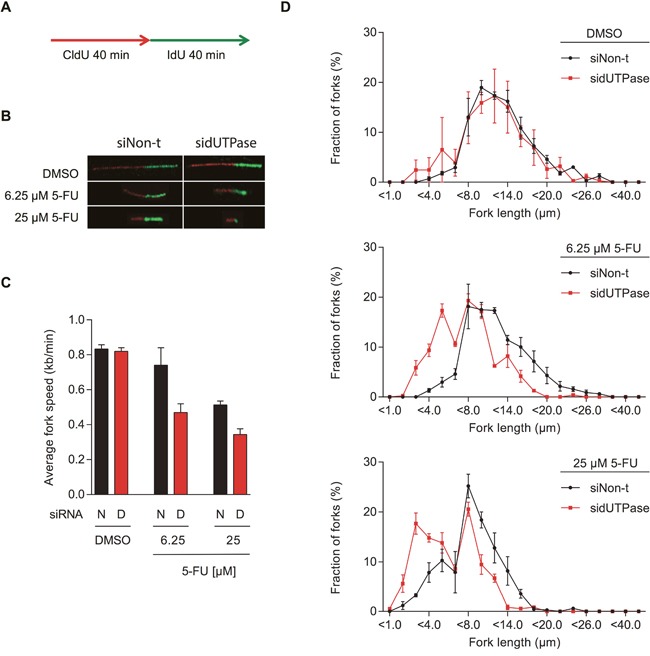
5-FU treatment decreases replication fork speed, which is enhanced by dUTPase depletion **(A)** Schematic illustration of CldU (red) and IdU (green) labeling during the DNA fiber assay. **(B)** Representative images of DNA replication fibers from dUTPase depleted and control cells treated with the indicated concentration of 5-FU. **(C)** Average fork speed during IdU labeling in dUTPase depleted and control cells after 48 hours of 5-FU treatment. Data shown as average ± SEM from three independent experiments. Abbreviations: N: siNon-t, D: sidUTPase. **(D)** Distribution of IdU labeled fiber length in dUTPase depleted and control cells. Data shown as average ± SEM from three independent experiments.

While the ADU technique evaluates the average replication speed in a defined population of cells, the EdU technique averages the EdU incorporation per cell. Nevertheless, these average values of replication speed could both indicate a reduced number of fired replication origins (with similar replication speed) or a reduction in fork progression. The speed of single replication forks can be analyzed by using the DNA fiber assay, in which successive incorporations of the thymidine analogues CldU and IdU into DNA is visualized by immunostaining (Figure 3A). Using this technique, we demonstrate that 5-FU treatment reduces the replication fork speed, explaining the accumulation of cells in S-phase (Figure 3A-3D). Depletion of dUTPase further decreases the DNA fiber lengths, demonstrating that individual replication forks are severely affected by lack of the dUMP substrate for TS.

### Characterization of the dUTPase inhibitors 1 and 2

**Figure 4:**
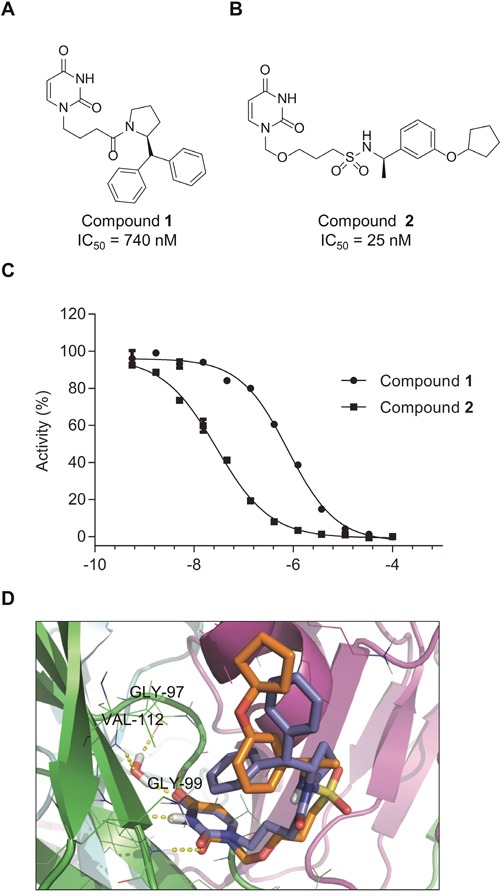
Compounds 1 and 2 inhibit dUTPase activity Chemical structures of the dUTPase inhibitors **1 (A)** and **2 (B) (C)** Potency of compounds **1** and **2** was assessed by malachite green assay, using dUTP as a substrate. Percentage activity was calculated compared to DMSO treated control. Inhibition curves shown are representative curves of two independent inhibition experiments performed using duplicate measurements. IC_50_ values displayed in **(A)** and **(B)** were determined from two independent experiments performed in duplicate and are shown as average ± SD. (D) Superposition of the top-ranked docking poses of compounds **1** (blue sticks) and **2** (orange sticks). The different monomers of the protein are rendered as green, cyan and magenta cartoons. H-bonds are shown as yellow dotted lines.

In order to study the effects of pharmacological dUTPase inhibition, two dUTPase inhibitors (compounds **1** and **2**, Figure 4A and 4B respectively) were synthesized as described in the Supplementary Materials and Methods (Supplementary Figure 2) [25–27].

The potency of these inhibitors was assessed using an *in vitro* activity assay, in which dUTPase catalyzed the hydrolysis of dUTP to dUMP and pyrophosphate (PPi). The conversion of inorganic phosphate (Pi) from PPi was analyzed using the malachite green reagent. For this purpose, dUTPase was expressed and purified from bacterial lysates (Supplementary Figure 3A) and its activity was assessed with dUTP and 5-FdUTP (6.6 and 5.5 μM formed PPi per second per μM enzyme, similar to previously reported data) (Supplementary Figure 3B) [28]. Compound **1** shows an IC_50_ of 740 nM while compound **2** exhibits an approximately 30-fold higher efficacy with an IC_50_ of 25 nM (Figure 4C). In addition, these compounds showed high selectivity in a pannel of various nucleoside triphosphate pyrophosphatase or phosphohydrolase enzymes tested (Supplementary Figure 4).

Computational docking predicts the putative binding modes for compounds **1** and **2** in the substrate binding pocket of dUTPase (Figure 4D and Supplementary Materials and Methods). For both compounds, the docking with the lowest Glide SP scores (−8.23 and −7.13 kcal/mol, respectively) had their uracil moieties inserted deep into the uracil binding pocket and displayed the same H-bonding patterns as described for other uracil-based ligands, interacting with Gly99, Gly110 and a conserved water molecule that bridges uracil, Gly97 and Val112. The flexible side-chains of both ligands had adopted U-shaped conformations with one of the aryl group folding back over the uracil moiety. The amide linker of inhibitor **1** and sulfonamide linker of compound **2** are facing the solvent, while the terminal cyclopentyl moiety of compound **2** partially occupies the same region as the second terminal phenyl group of compound **1**. The benzylic α-methyl group of inhibitor **2** occupied the same region as the proline ring of compound **1**, providing a hypothesis for the observed stereochemical preference displayed by structurally closely related representatives of these two chemical series [29, 30].

### dUTPase inhibitors sensitize colorectal cancer cells to 5-FU treatment

**Figure 5:**
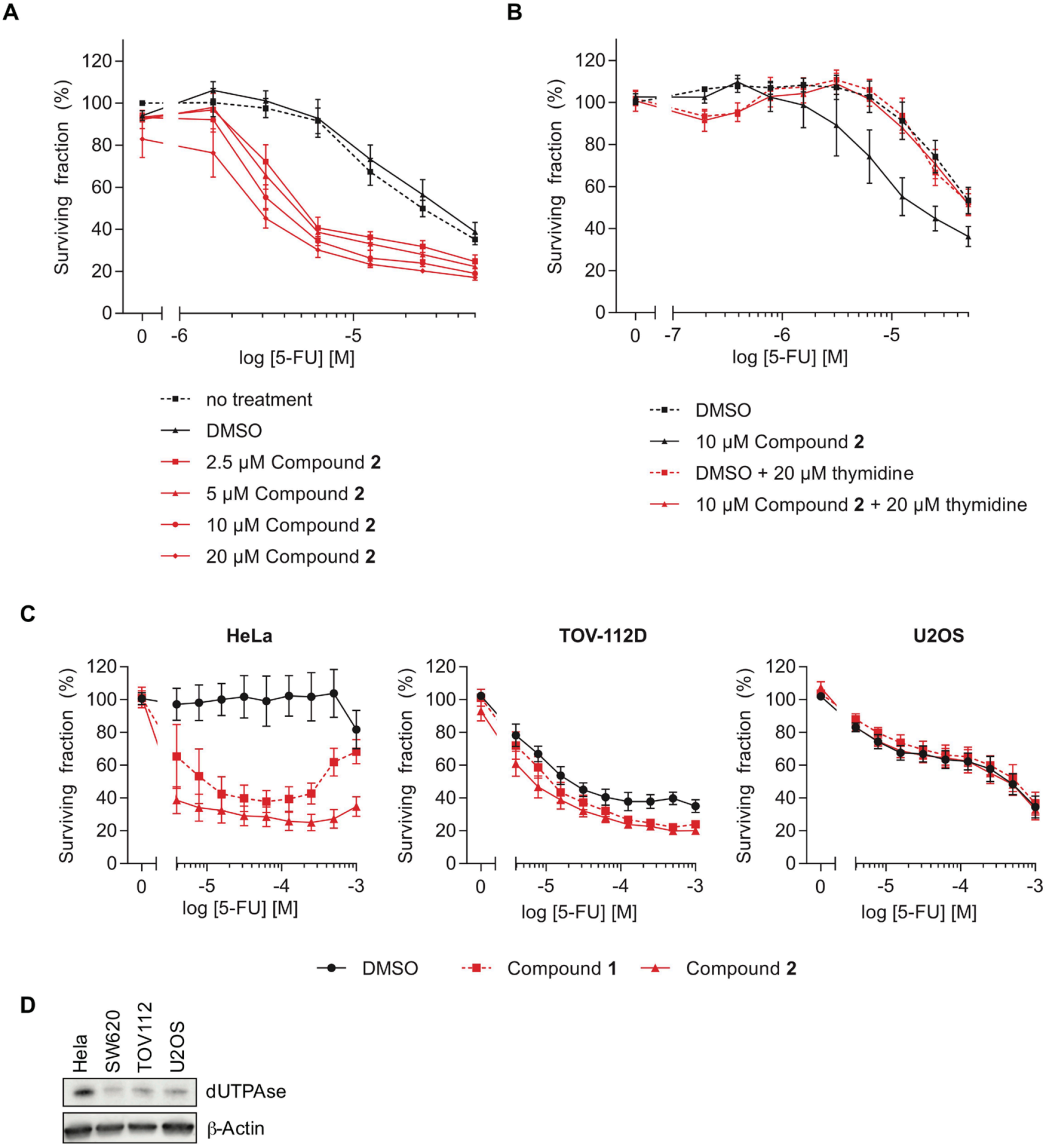
The dUTPase inhibitors sensitize cells to 5-FU treatment **(A)** Resazurin experiment assessing the viability of SW620 cells co-treated with 5-FU and the dUTPase inhibitor **2** at the indicated concentrations for 72 hours. Values were normalized against the no-DMSO control (−). Data shown as average ± SEM from three independent experiments performed in duplicate. **(B)** Resazurin experiment analyzing cellular viability after 72 hours of co-treatment with 5-FU and compound **2** (10 μM) in combination with thymidine (20 μM). Values were normalized against the DMSO control. Data shown as average ± SEM from at least four independent experiments performed in duplicate. **(C)** Viability of HeLa, TOV-112D and U2OS cells treated with 10 μM of compound **1** or **2** and increasing concentrations of 5-FU. After 72 hours of the indicated treatment cell viability was analyzed by Resazurin. Data shown as average ± SEM from at least three independent experiments performed in duplicate. **(D)** Western Blot analyzing the dUTPase levels in the indicated cell lines. β-Actin was used as a loading control.

We next analyzed whether pharmacological inhibition of dUTPase, using compounds **1** or **2**, is a potential strategy to increase the efficacy of 5-FU. Cell survival was assessed after 72 hours of co-treatment using the resazurin assay. Inhibition of dUTPase significantly increased the cytotoxicity of the 5-FU treatment (Figure 5A and Supplementary Figure 5A for compounds **2** and **1**, respectively). In line with protein depletion, dUTPase inhibition alone did not induce cellular toxicity at the concentrations and time points tested. Importantly, the toxicity induced by **2** upon 5-FU treatment was rescued by addition of thymidine (Figure 5B). In addition, the cervix cancer cell line HeLa showed increased sensitivity to 5-FU by addition of compounds **1** or **2** and to a minor extend a slight effect is observed with the cell line TOV-112D (ovary origin) (Figure 5C). On the contrary, the osteosarcoma cell line U2OS showed no increase in 5-FU toxicity when dUTPase was inhibited. The sensitivity did only partially correlate with dUTPase expression levels, as both HeLa and SW620 cells exhibit increased sensitivity to 5-FU upon addition of compounds **1** or **2**, but only Hela cells show high expression levels of dUTPase (Figure 5D and Supplementary Figure 7B). These data demonstrate a variability in potentiation of 5-FU toxicity in different cancer cell lines.

### dUTPase inhibitors increase 5-FU induced replication defects and DNA damage

**Figure 6:**
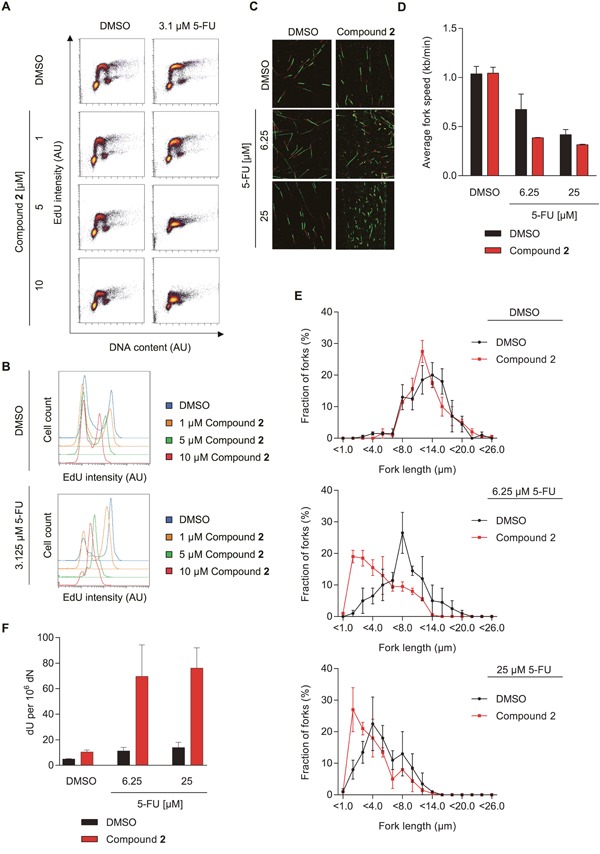
The dUTPase inhibitors increase 5-FU efficacy by increasing dU in DNA and reducing replication fork speed **(A)** FACS analyses measuring the EdU incorporation of SW620 cells treated for 48 hours with 3.1 μM of 5-FU in combination with increasing doses of compound **2**. EdU intensity is depicted on the y-axis and DNA content (To Pro intensity) on the x-axis. A representative experiment is shown. **(B)** Representative histograms displaying the EdU intensity of the experiment shown in Figure 6A. The intensity of the PI staining is depicted on the x-axis and events (cell count) on the y-axis. Abbreviation: AU: arbitrary unit. **(C)** Representative images of DNA replication forks from cells treated with the indicated concentration of 5-FU or DMSO in combination with 10 μM of compound **2** or a DMSO control for 48 hours. **(D)** Average fork speed during IdU labelling of cells treated as described in Figure 6C. Data shown as average ± SEM from two independent experiments. **(E)** Distribution of IdU labelled fiber length of the experiment shown in Figure 6C–6D. Data shown as average ± SEM from two independent experiments. **(F)** dU content in SW620 cells treated for 48 hours with the indicated concentration of 5-FU in combination with compound **2** or DMSO, by mass spectrometry. Data shown as average ± SEM from three independent experiments.

We then studied the effects of the dUTPase inhibitors on 5-FU-induced S-phase arrest by co-treating cells for 48 hours with inhibitor **2** and 5-FU and subsequent labelling with EdU. In cells treated with 3.1 μM of 5-FU, inhibition of dUTPase by compound **2** further reduced the amount of incorporated EdU in a dose dependent manner (Figure 6A-6B). DNA fiber experiments revealed that the reduced EdU incorporation also correlated with decreased replication fork progression, supporting the data previously obtained by dUTPase knockdown experiments (Figure 6C-6E and Supplementary Figure 5B-5D).

Staining of phosphorylated histone H2A.X (γH2AX) is commonly used to visualize DNA damage in association with replication fork stress [31]. Here, we determine γH2AX foci formation by automated microscopy following treatment of cells for 72 hours with 5-FU, and demonstrate that addition of compound **1** or **2** to the 5-FU treatment further increased DNA damage (Supplementary Figure 6). No increase in γH2AX foci could be detected in cells treated with the dUTPase inhibitors alone.

dUTPase inhibition likely leads to accumulation of dUTP and 5-FdUTP and subsequent misincorporation into DNA. To test this hypothesis, we analyzed dU and 5-FdU levels in DNA using mass spectrometry. While 5-FU treatment alone had only minimal effects on the dU levels in DNA, simultaneous dUTPase inhibition significantly raised the amount of dU incorporation into DNA (Figure 6F). Following co-treatment of compound **2** and 5-FU, some low levels of 5-FdU in DNA were observed but were too close to detection limit to make any firm conclusion (data not shown). No 5-FdU in DNA was detected in DNA from single-treated cells (data not shown).

## DISCUSSION

Even 60 years after the first synthesis of antifolates and fluoropyrimidines, the complex mechanism of action is still debated [32, 33]. Initially, depletion of thymidine was thought to be the main cause of 5-FU induced toxicity [34, 35]. Many studies have in addition highlighted the importance of elevated levels of both uracil and 5-fluorouracil and their misincorporation into DNA [6, 36, 37]. More recently, incorporation of 5-FUTP into RNA and its associated transcription defects have been considered as the main cause of cell death [38]. Studying the mechanism of action of 5-FU is necessary to understand and overcome frequently observed drug resistance and ultimately improve patient care.

Here, we investigated the DNA replication defects induced by 5-FU treatment and the importance of 5-FdUTP accumulation for this effect. By analyzing EdU incorporation into DNA, we observed that 5-FU treatment leads to accumulation of cells in the S-phase of the cell cycle. ADU experiments and DNA fiber analyses demonstrated slower DNA replication fork progression upon 5-FU treatment, which are in agreement with low dTTP levels generated by TS inhibition. Hence, our conclusion is that reduced replication fork speed by combination treatment of dUTPase inhibitors/siRNA and 5-FU is a result of even lower levels of dTTP, caused by low substrate dUMP levels (by dUTPase loss) and low TS activity (by 5-FU treatment). Uracil analogues (EdU, CldU, IdU) were used for the DNA fiber and cell cycle experiments. Since these uracil analogues are already modified on the 5′-position they likely do not need dUTPase activity to be incorporated into DNA as also suggested by the fact that we observed no decrease in the intensity of fibers following dUTPase inhibition or siRNA treatments.

Interestingly, protein depletion and pharmacological inhibition of the nucleotide triphosphatase dUTPase further augmented the amount of uracil in DNA, DNA replication defects, DNA damage and cytotoxicity of 5-FU, highlighting the importance of (5-F)dUTP accumulation for cytotoxicity. However, one should keep in mind that the 5-FU metabolism involves various enzymes and intermediate species and that the mode of toxicity is most likely multifaceted and dependent on the molecular makeup of the cell.

Despite this complexity, a number of studies have shown that dUTPase levels significantly influence the efficacy of 5-FU and other TS-based therapies [17–20, 22, 39]. These studies have often used siRNA mediated dUTPase depletion. However, in certain situations a discrepancy between protein inhibition and depletion can be observed. Here, we showed that inhibiting dUTPase with small molecules leads to comparable effects as protein depletion by siRNA.

Furthermore, we show that inhibiting dUTPase activity, both by siRNA and pharmacological inhibition, does not lead to severe toxicity when used as a mono-treatment. A favorable safety profile was also confirmed by the phase I clinical trial of the dUTPase inhibitor TAS-114 [40].

Importantly, tumors were found to have dysregulated dUTPase expression and high nuclear dUTPase expression correlated with therapy resistance, shorter time to progression and shorter overall survival [21]. With this study, we further elucidate the mechanism of 5-FU-induced toxicity by investigating DNA replication defects. Inhibiting dUTPase activity by siRNA or inhibitors significantly augmented 5-FU induced replication defects and toxicity, highlighting the contribution of (5-F)dUTP to toxicity. These results demonstrate the high potential of dUTPase inhibitors to improve current TS therapies.

## MATERIALS AND METHODS

### Cell culture

SW620, HeLa, TOV-112D and U2OS cells were cultured in 37°C with 5% CO_2_ using DMEM (Life Technologies), supplemented with fetal calf serum (10%), penicillin (50 U/mL) and streptomycin (50 μg/mL). Mycoplasma contamination was assessed using the MycoAlert™ Mycoplasma Detection Kit (Lonza). Thymidine (Sigma-Aldrich) was diluted in H_2_O, 5-FU (Sigma-Aldrich) was diluted in DMSO to 200 mM, while compounds **1** and **2** were dissolved in DMSO to 10 mM. DMSO concentrations were adjusted to equal levels in all treatments.

### RNAi transfection

siRNA was transfected using INTERFERin® as suggested by the manufacturer's instructions (polyplus transfection™). Oligonucleotides targeting all three isoforms of dUTPase (sense strand: 5′CGGACAUUCAGAUAGCGCUTT-3′; antisense strand: 5′-AGCGCUAUCUGAAUGUCCGTT-3′; referred to as sidUTPase) and the All-stars negative control (referred to as siNon-t) were obtained from Qiagen and transfected to a final concentration of 10 nM. Cells were siRNA transfected for 48 hours, re-seeded and incubated overnight to achieve attachment. Additional treatment was performed as indicated in the different sections.

### Western blot analysis

Cells were lysed with RIPA buffer (150 mM NaCl, 1.0% NP-40, 0.5% sodium deoxycholate, 0.1% SDS, 50 mM Tris-HCl (pH 8.0), complete protease inhibitor cocktail (Roche) and phosphatase inhibitors (Phosphatase Inhibitor cocktail, Life Technologies)). After sonication, the debris was removed by centrifugation. Protein concentration was assessed using the Pierce™ BCA Protein Assay Kit (Thermo Fisher). Lysates, supplemented with 4x Laemmli buffer (Bio-Rad) containing 2-mercaptoethanol, were heated to 95°C for 5 min. Proteins were separated with Mini-PROTEAN TGX™ gels (Bio-Rad) using Tris/Glycine/SDS running buffer (Bio-Rad) and transferred to a 0.45 μm nitrocellulose membrane (Bio-Rad) with the Trans-Blot Turbo™ Transfer Starter System (Bio-Rad). Membranes were blocked with Odyssey Blocking Buffer (LI-COR, 1:1 in PBS) before the primary antibodies, anti-dUTPase (1:500; rat; a kind gift from Prof. Grässer [41]) and β-Actin (Abcam, ab6276), were added overnight at 4°C. IRDye® 800CW secondary antibodies anti-rat and anti-mouse (LI-COR) were added for 1 hour. Fluorescence was visualized using an Odyssey® Fc Imager and Image Studio™ Software (LI-COR). The specificity of the anti-dUTPase antibody is shown in Supplementary Figure 7.

### Clonogenic survival assay

After siRNA transfection, the indicated concentration of 5-FU was added for 48 hours, followed by a 24 hours recovery period with fresh media. 200 cells were re-seeded onto petri dishes in triplicate and incubated for 10 days. Colonies were fixed and visualized using methylene blue (4 g/L) in methanol and then assessed by eye. Surviving fractions were calculated by averaging the triplicate values and normalizing these against the untransfected DMSO control.

### Propidium iodine (PI) FACS analysis

Following the indicated treatment, the cells and the media were collected. Samples were washed and fixed by freezing cells in 70% ethanol. After two PBS washes, 0.5 mL PI solution (25 μg/mL PI (Sigma) and 100 μg/mL RNaseA (Thermo Fisher Scientific) in PBS) was added for 20 min. PI intensity was measured on a FACSCalibur (Becton Dickinson) and the cell cycle was analyzed using WinMDI 2.9.

### 5-ethynyl-2′-deoxyuridine (EdU) and To Pro FACS analyses

To assess replication, cells were pulse labeled using 1 μM EdU for 30 min. The Click-iT® EdU Alexa Fluor® 488 Imaging Kit (Molecular Probes) was used as described in the manufacturer's manual. DNA was counterstained with 1 μg/mL To Pro (Molecular Probes). EdU and To Pro intensities were measured on a FACSCalibur (Becton Dickinson) and analyzed using WinMDI 2.9 and Cytobank.

### Alkaline DNA unwinding (ADU) technique

The method was performed as described by Johansson *et al*. [42]. Briefly, cells were pulse-labeled with ^3^H-thymidine (7.4 kBq/mL; GE Healthcare) for 30 min. Cells were washed and incubated in media plus the indicated treatment for the specified time-points. Ice-cold 0.03 M of NaOH in 0.15 M of NaCl was added for 30 min incubation on ice and in darkness. Addition of 1 mL of 0.02 M NaH_2_PO_4_ stopped the unwinding. The DNA was fragmented by ultrasonic treatment for 15 seconds (B-12 sonifier with micro-tip; Branson). SDS was added to a final concentration of 0.25% and samples were frozen overnight. After a 1:1 dilution with distilled water, the samples were added to hydroxyapatite columns mounted in an aluminum block maintained at 60°C. The columns were washed with 0.5 M potassium phosphate before the single stranded and then double stranded DNA fractions were respectively eluted with 0.1 M and 0.25 M potassium phosphate buffer. Radioactivity was assessed on a RackBeta scintillation counter. The amount of single stranded DNA was compared to the total labeled DNA.

### DNA fiber technique

The DNA fiber technique was similarly performed as described by Groth *et al*. [43]. Cells were treated as indicated before, 5-chloro-2′-deoxyuridine (CldU) (25 μM; Sigma) was added for 40 min followed by 40 min incubation with 5-iodo-2′-deoxyuridine (IdU) (250 μM; Sigma), with the indicated treatment present. Cells were scraped in ice-cold PBS. Unlabeled and labeled cells were mixed in equal proportions. 2.5 μL of the cell suspension were mixed with 7.5 μL spreading buffer (200 mM Tris-HCl, pH 7.4, 50 mM EDTA and 0.5% SDS) on microscopy slides (SuperFrost®, Menzel Gläser, VWR). After 8 min, the slides were tilted to spread the DNA and then fixed by incubation in MeOH/AcOH (3:1) overnight at 4°C. Samples were denatured in 2.5 M HCl for 1 hour and unspecific binding was blocked using PBS containing 1% BSA and 0.1% Tween20. For immunodetection of CldU and IdU, the slides were incubated with monoclonal rat anti-BrdU Ab (Clone BU1/75 (ICR1); Oxford Biotechnologies) and monoclonal mouse anti-BrdU Ab (Clone B44; Becton Dickinson, 347580). Anti-rat Alexa Fluor® 555 and anti-mouse Alexa Fluor® 488 (1:500 in blocking solution; Life Technologies) were used as secondary antibodies. Images of coded samples were taken on a Zeiss LSM 510 or 780 inverted confocal microscope. Fiber length was measured using the ImageJ software. 1 μm was converted to 2.59 kilo base pairs. At least 100 forks were analyzed per sample.

### Resazurin survival assay

2000 cells were seeded in 50 μL medium per well into 96-well plates. 24 hours later, 40 μL of compound **1** or **2** was added to reach a final concentration of the indicated dose (after addition of 10 μL 5-FU stock). After 2 hours, 10 μL of the 5-FU stock was added to each well to reach the indicated concentration. After 72 hours, resazurin was added to a final concentration of 10 μg/mL and the cells were incubated 3 hours. Fluorescence intensity was measured at 544/590 nm (Ex/Em). Relative survival of the cells was calculated by subtracting the background fluorescence, averaging duplicate measurements and normalizing the value to the untreated well.

### Quantification of modified bases in genomic DNA

DNA for nucleoside quantification was isolated by phenol:chloroform:isoamyl alcohol extraction as previously described [44]. Cells were lysed by passing through 21G and 23G syringe needles and subsequent incubation at 37°C for 1 h with 1000 RPM shaking in a buffer containing 10 mM Tris-HCl (pH 8.0), 10 mM NaCl, 1% SDS, 100 mM DTT, 0.1 mg/mL proteinase K (Worthington Biochemical), 0.1 mg/mL RNase A (Sigma-Aldrich), 50 μM tetrahydrouridine (THU, Merck Millipore). DNA was subsequently extracted from the lysates with 25:24:1 phenol:chloroform:isoamyl alcohol, followed by two washes with 24:1 chloroform:isoamyl alcohol and isopropanol precipitation using 10 M ammonium acetate to precipitate the DNA. RNA and free nucleotides were then removed from the DNA samples by treatment with 4 μg RNase A in 10 mM ammonium bicarbonate (pH 7.0), 10 mM MgCl_2_ for 30 min at 37°C, followed by a subsequent isopropanol precipitation.

Next, the DNA samples were hydrolyzed and dephosphorylated to single nucleosides as previously described [44]. DNA was hydrolyzed to nucleosides by treatment with 0.8 U Nuclease P1 (Sigma-Aldrich), 80 U Benzonase (Santa Cruz Biotechnology), and 7.5 U Antarctic Phosphatase (New England Biolabs) in 50 μL reactions containing 10 mM ammonium acetate (pH 5.5), 1 mM MgCl_2_, 0.1 mM ZnCl_2_ and 240 μM THU for 60 min at 37°C. Enzymes were then precipitated and removed from the reactions by adding three volumes of ice-cold acetonitrile to the reactions, incubating for 10 min on ice, centrifugation at 16,100 rcf for 30 min at 4°C. The supernatants were transferred to new tubes and lyophilized until dry. For 5-fluoro-2´-deoxyuridine (5FdU), 0.2 U alkaline phosphatase (Sigma-Aldrich) and 240 μM Deferoxamine mesylate (Santa Cruz Biotechnology) were used instead of Antarctic Phosphatase and THU.

To separate dU from dC, the samples were redissolved in water and fractionated on an Agilent 1100 HPLC system (with a UV detector set to 260 nm to identify the canonical nucleosides) and a mixed mode Primesep 200 column (2.1 mm x 150 mm, 5 μm, SieLC) kept at 30°C using a flow rate of 0.4 mL/min and water and acetonitrile as mobile phase, each containing 0.1% formic acid, as the mobile phase. The 12-min-long HPLC gradient was as follows: 5% acetonitrile for 30 s, ramp to 35% acetonitrile by 1.5 min to 2.5 min, and return to 5% acetonitrile by 2.51 min. The dU-containing fractions were collected from 1.6-1.7 min and vacuum centrifuged until dry. Samples were not pre-fractionated for 5-FdU analysis. The pellets were redissolved in water and analysed by LCMS/MS using a reverse phase column (2.1 mm x 150 mm, 1.8 μm, EclipsePlusC18 RRHD, Agilent Technologies) kept at 25°C with a flow rate of 0.3 mL/min on a 1290 Infinity II HPLC coupled to a 6495 Triple Quadrupole mass spectrometer with an electrospray ion source (Agilent Technologies). Water and methanol were used as the mobile phase, each containing 0.1% formic acid. The 13-min-long HPLC gradient was as follows: 5% methanol for 3 min, ramp to 13% methanol by 3.5 min, ramp to 17% methanol by 5.5 min to 7 min, and return to 5% methanol by 8 min. Analysis was performed in positive ionization multiple reaction monitoring mode, using the mass transitions 229.08 → 113.0, 232.08 → 116.0, and 247.1 → 131.0 for 2´-deoxyuridine (dU), ^13^C^15^N_2_-dUrd, 5-FdU, respectively.

The Supplementary Materials and Methods contain additional information regarding the expression and purification of human recombinant dUTPase, the dUTPase activity and inhibition assay, the detailed synthetic route for dUTPase inhibitors **1** and **2**, as well as their molecular dockings with dUTPase, and the analysis of phosphorylated H2A.X.

## SUPPLEMENTARY MATERIALS FIGURES


