# Supplementary material for: dUTPase inhibition augments replication defects of 5-Fluorouracil
Source: Oncotarget. 2017 Feb 28;8(14):23713–26. doi: 10.18632/oncotarget.15785 (PMC5410339; doi:10.18632/oncotarget.15785)
Supplement: Supplementary file 1 [file oncotarget-08-23713-s001.pdf]

dUTPase inhibition augments replication defects of 5-Fluorouracil

SUPPLEMENTARY MATERIALS AND METHODS

Expression and purification of human recombinant dUTPase

cDNA encoding human dUTPase, codon optimized for expression in *E. coli,* was purchased from GeneArt (Invitrogen) and was subcloned into the expression vector pET28a (Novagen) using NdeI and NotI restriction sites. The expression construct (pET28a-dUTPase) was transformed into the *E. coli* strain BL21(DE3). The transformed cells were grown in LB medium at 37 °C to OD_{600} of 0.7 when dUTPase expression was induced by 1 mM IPTG for 3 hours. Cells were harvested by centrifugation and the obtained pellet was dissolved in B-PER bacterial protein extraction reagent (Pierce) with complete protease inhibitor cocktail (Roche) and Benzonase nuclease (Millipore). The suspension was centrifuged and the cleared supernatant was subjected to chromatography on Ni-Sepharose column HisTrap HP (GE Healthcare). The column was pre-equilibrated with 100 mM Tris-HCl buffer (pH 7.7, containing 250 mM NaCl, 50 mM imidazole, and 5 % glycerol) and proteins were eluted by a linear imidazole gradient (50 mM – 500 mM) in the same buffer. Fractions containing dUTPase were dialyzed against 20 mM Tris-HCl (pH 8.5), 20 mM NaCl, and 5 mM $MgCl_2$ buffer, loaded onto a MonoQ anion exchange column (HiTrap HP, GE Healthcare) and proteins were eluted with a NaCl gradient (20 mM – 1000 mM) in the same buffer. The purity of the final fraction of His-tagged dUTPase was assessed by Sodium dodecyl sulphate (SDS)-PAGE. Protein concentration was determined by Coomasie Plus protein assay kit (Pierce) with BSA as a standard. The fractions with purified dUTPase were combined, dialyzed against 20 mM Tris-HCl (pH 8.5), 150 mM NaCl, and 5 mM $MgCl_2$, and kept at -80 °C.

dUTPase activity and inhibition assay

Activity of human dUTPase was tested in assay buffer (100 mM TrisAcetate (pH 8.0), 40 mM NaCl, 10 mM MgAc, 1 mM DTT and 0.005% Tween-20). dUTPase (9 nM) was incubated with 50 µM dUTP or 50 µM 5-FdUTP for 15 minutes at 22 °C in the presence of an excess of *E. coli* PPase (0.2 U/mL) converting produced pyrophosphate (PPi) to phosphate (Pi). Formed Pi was detected by the addition of a malachite green reagent [1] and after a 15 minutes incubation with shaking, the absorbance was read at 630 nm using a Hidex plate reader. Each assay point was performed at least in duplicate. A Pi standard curve was included to convert absorbance to concentration Pi.

For IC_{50} value determination, a 12 point serial dilution of compounds **1** and **2** with three-fold difference between each concentration was generated, using 100 µM as the highest concentration. dUTPase (4.5 nM) was incubated with 12.5 µM dUTP and the indicated concentration of compounds **1** or **2** for 15 minutes at 22 °C in assay buffer fortified with *E. coli* PPase as described above. DMSO was used as control. Formed Pi was measured as described above. Percent activity was calculated and plotted against log[inhibitor]/M and IC_{50} values were determined by fitting the equation "log[inhibitor] *vs*. response - Variable slope" in the GraphPad Prism software to the data.

Synthesis of dUTPase inhibitors 1 and 2 (Supplementary Figure 4)

Compound **1** was obtained *via* a three-step sequence involving selective alkylation of N_1-uracil, saponification of the ester motif and amidation [2, 3].

Taking inspiration from Taiho's procedure [4], compound **2** was synthesized from chiral amine **5** in 6% overall yield over 10 steps. After Boc-protection of amine **5**, a palladium-catalyzed coupling afforded pinacol-boronic ester intermediate **7**, which was oxidized in the presence of hydrogen peroxide to give chiral phenol **8**. Alkylation of **8** was followed by a Boc-deprotection under acidic conditions. After formation of the sulfonamide **11** from amine **10**, the chlorine motif was converted into the corresponding ester **12**, which in turn was hydrolyzed into alcohol **13** under acidic conditions. Finally, freshly prepared silyl enol ether was coupled to activated uracil to afford compound **2**.

General method for the synthesis of dUTPase inhibitors 1 and 2

All reagents were commercial grade and used without further purification. Flash column chromatography was performed on Merck silica gel 60 (70-230 mesh). Preparative HPLC was performed on a Gilson HPLC system: Column ACE 5 C8 (150330 mm); Water (containing 0.1% TFA) and acetonitrile were used as mobile phases. Analytical LCMS was conducted using an Agilent MSD mass spectrometer connected to an Agilent 1100 HPLC. System A: Column ACE 3 C8 (503 3.0 mm); Water (containing 0.1% TFA) and acetonitrile were used as mobile phases; system B: Xterra MS C18

(5033.0 mm); Water (containing 10 mM NH_4HCO_3; pH: 5-10) and acetonitrile were used as mobile phases. All compounds gave satisfactory purities when analysed using both systems. ^{1}H NMR spectra were recorded on a Bruker Advance DPX 400 spectrometer at 400.1 MHz. For NMR experiments recorded in deuterated chloroform, the resonances of the residual solvent peaks at 7.27 ppm for the ^{1}H spectra were used as internal references. For NMR experiments recorded in deuterated methanol, the resonances of the residual solvent peaks at 3.35 ppm for the ^{1}H spectra were used as internal references. Chemical shifts (δ) are measured in ppm and coupling constants (*J*) are quoted in Hz to one decimal place. Spectral data for ^{1}H NMR spectra are reported as follows: chemical shift (multiplicity, number of protons, coupling constants). In reporting spectral data, the following abbreviations are used for multiplicity in ^{1}H NMR spectra: s (singlet), d (doublet), dd (doublet of doublets), dt (doublet of triplets), ddd (doublet of doublet of doublets), t (triplet), m (multiplet), br (broad), app (apparent), q (quadruplet), quint (quintuplet). In the case of ambiguous assignments, 2-dimensional homonuclear (^{1}H-^{1}H) and heteronuclear (^{1}H-^{13}C) NMR experiments were recorded. Instant JChem was used for structure and reagent database management, Instant JChem 6.1.0, 2013, ChemAxon (http://www.chemaxon.com).

Synthesis of dUTPase inhibitor 1

Synthesis of ethyl 4-(2,4-dioxo-3,4-dihydropyrimidin-1(2H)-yl)butanoate 3

Ethyl-4-bromobutyrate (390 mg, 2.0 mmol, 300 µL) was added to a suspension of uracil (291 mg, 2.6 mmol) and caesium carbonate (717 mg, 2.2 mmol) in dry dimethylformamide (7 mL). The resulting suspension was stirred at room temperature overnight. After completion of the reaction, most of the dimethylformamide was evaporated and water (20 mL) and EtOAc (20 mL) were added to the residue. The phases were separated and the aqueous phase was extracted with EtOAc (2 × 30 mL). The combined organic layers were washed with water (3 × 10 mL), dried over $MgSO_4$ and evaporated. The residue was purified by flash chromatography (eluent: EtOAC/^{i}Hex 2/1) to afford the desired product **3** as a colorless oil (146 mg, 32%). **ESI-MS*:*** m/z = 228 [M+H]$^+$. **^{1}H NMR** (400 MHz, $CDCl_3$) δ 1.29 (t, 3H, *J* 7.3), 2.04 (quint, 2H, *J* 7.0), 2.40 (t, 2H, *J* 7.0), 3.82 (t, 2H, *J* 7.3), 4.17 (q, 2H, *J* 7.3), 5.72 (d, 1H, *J* 7.9), 7.22 (d, 1H, *J* 7.9),8.49 (br s, 1H).

Synthesis of *(S)*-1-(4-(2-benzhydrylpyrrolidin-1-yl)-4-oxobutyl)pyrimidine-2,4(1H,3H)-dione 1

Ester **3** (57 mg, 0.25 mmol) was dissolved in methanol (2 mL) and treated with 1M $NaOH_{aq}$ (0.5 mL). The reaction was stirred at room temperature for 2h. The mixture was acidified using 2M HCl_{aq}, the solvent was evaporated and the residue was dried under vacuum. Acid **4** was used in the next step without further purification.

To a suspension of acid **4** (36 mg, 0.18 mmol), hydroxybenzotriazole (29.5 mg, 0.22 mmol) and 1-ethyl-3-(3-dimethylaminopropyl)carbodiimide (33.8 mg, 0.22 mmol) in dimethylformamide (1.5 mL) was added triethylamine (74 µL). The reaction was stirred for 10 minutes at room temperature prior to the addition of (*S*)-(-)-2-(diphenylmethyl)pyrrolidine (53.8 mg, 0.2 mmol). The reaction was stirred overnight at room temperature. The mixture was concentrated prior to purification by flash chromatography. The desired product **1** was obtained as a light orange solid (35 mg, 46%). **ESI-MS**: m/z = 419 [M+H]$^+$. Presumed mixtures of rotamers (2:1). **^{1}H NMR** major product (400 MHz, $CDCl_3$) δ 1.47-1.70 (m, 2H), 1.75-1.98 (m, 5H), 2.15-2.30 (m, 1H), 3.21-3.53 (m, 4H), 3.80-3.83 (m, 1H), 3.95 (d, 1H, *J* 10.1), 4.48 (dd, 1H, *J* 10.1, 6.1), 5.63-5.67 (m, 1H), 7.09-7.38 (m, 11H),8.85 (br s, 1H).

^{1}H NMR minor product (400 MHz, $CDCl_3$) δ 1.47-1.70 (m, 2H), 1.75-1.98 (m, 5H), 2.15-2.30 (m, 1H), 3.21-3.53 (m, 4H), 3.62-3.70 (m, 1H), 4.37 (d, 1H, *J* 7.3), 5.13 (td, 1H, *J* 7.3, 2.2), 5.63-5.67 (m, 1H), 7.06 (d, 1H, *J* 7.9), 7.09-7.38 (m, 10H),8.83 (br s, 1H).

Synthesis of dUTPase inhibitor 2

Synthesis of *(R)*-*tert*-butyl (1-(3-bromophenyl)ethyl) carbamate 6

A suspension of the amine **5** (5.0 g, 0.025 mol) and sodium bicarbonate (3.36 g, 0.04 mol) in methanol (200 mL) was treated portion-wise with di-*tert*-butyl dicarbonate (5.67 g, 0.026 mol) and stirred at room temperature for 3 hours. The methanol was removed by evaporation. The residue was treated with water (50 mL), then extracted with ethyl acetate (2 x 40 mL). Evaporation of the extract gave the desired product **6** as a colorless gum (7.70 g, quantitative yield). **^{1}H NMR** (400 MHz, $CDCl_3$) δ 1.42 (s, 9H), 1.43 (overlapping d, 3H, *J* 6.8), 4.77 (br s, 2H), 7.20 (app t, 1H, *J* 7.7, 7.5,), 7.23 (dt, 1H, *J* 7.7, 1.5), 7.38 (dt, 1H, *J* 7.5, 1.8), 7.45 (app t, 1H, *J* 1.8, 1.5).

Synthesis of *(R)*-*tert*-butyl (1-(3-(4,4,5,5-tetramethyl-1,3,2-dioxaborolan-2-yl)phenyl)ethyl)-carbamate 7

A suspension of the aryl bromide **6** (3.00 g, 10 mmol) *bis*(pinacolato)-diboron (3.80 g, 15 mmol), potassium acetate (2.45 g, 25 mmol) and [1,1′-*bis*(diphenylphosphino)-ferrocene] dichloropalladium(II), complex with dichloromethane (0.41 g, 0.5 mmol) in dioxane (50 mL) was heated at 80 °C overnight. The mixture was filtered through a plug of silica and the filtrate evaporated. The residue was purified by flash-chromatography over silica (eluent: ^{i}Hex/EtOAc 90/10) to afford the desired product **7** as a colorless gum (3.9 g, quantitative yield). **^{1}H NMR** (400 MHz, $CDCl_3$) δ

1.27 (br s, 12H), 1.42-1.49 (m, 12H), 4.62 (br s, 2H), 7.34 (app t, 1H, *J* 7.5, 7.3), 7.40 (br d, 1H, *J* 7.5), 7.75 (dt, 1H, *J* 7.3, 1.3), 7.74 (br s, 1H).

Synthesis of *(R)-tert*-butyl (1-(3-hydroxyphenyl)ethyl) carbamate 8

A stirred solution of the boronic ester **7** (3.74 g, 0.011 mol) in methanol (100 mL) was cooled to 0 °C then treated dropwise with 35% (w/v) aqueous hydrogen peroxide. The mixture was then allowed to warm to room temperature and stirred for an additional 45 minutes. The mixture was then cooled (ice-bath) and quenched with 0.03 M aqueous sodium thiosulphate (30 mL). The mixture was concentrated to remove most of the methanol then extracted with ethyl acetate (15 mL). Evaporation of the extract gave a pale yellow gum which was purified by flash-chromatography over silica (eluent: ^{i}Hex/EtOAc 80/20) to afford the desired product **8** (2.67 g, quantitative yield). ^{1}**H NMR** (400 MHz, $CDCl_3$) δ 1.41-1.44 (m, 12H), 4.73 (br s, 1H), 4.85 (br s, 1H), 6.07 (br s, 1H), 6.71 (ddd, 1H, *J* 8.0, 2.5, 0.8), 6.76 (br s, 1H), 6.83 (d, 2H, *J* 7.7), 7.17 (app t, 1H, *J* 8.0, 7.7).

Synthesis of *(R)-tert*-butyl (1-(3-(cyclopentyloxy) phenyl)ethyl)carbamate 9

Phenol **8** (2.67 g, 11.25 mmol), cyclopentyl bromide (2.41 mL, 22.50 mmol) and caesium carbonate (7.33 g, 22.50 mmol) were dissolved in dimethylformamide (20 mL). The reaction mixture was stirred at 80 °C for 48 hours. The salts were filtered off and the solvent was removed under reduced pressure. The residue was partitioned between CH_2Cl_2 and a saturated solution of $NaHCO_3$. The phases were separated and the organic layer was washed successively with NaCl, water and dried over Na_2SO_4. The resulting organic layer was concentrated under reduced pressure to afford the desired product **9** (2.89 g, 84%) as a yellow solid. ^{1}**H NMR** (400 MHz, $CDCl_3$) δ 1.42 (s, 9H), 1.42-1.44 (overlapping d, 3H, *J* 6.8), 1.56-1.66 (m, 2H), 1.78-1.94 (m, 6H), 4.73-4.77 (m, 3H), 6.75 (ddd, 1H, *J* 8.0, 2.2, 0.8), 6.81 (t, 1H, *J* 2.2), 6.85 (d, 1H, *J* 7.8), 7.22 (app t, 1H, *J* 8.0, 7.8).

Synthesis of *(R)*-1-(3-(cyclopentyloxy)phenyl) ethanamine, hydrochloride salt 10

To a solution of protected amine **9** (2.80 g, 9.17 mmol) in dioxane (10 mL) was added HCl 4N in dioxane (1.85 mL, 7.39 mmol). The reaction mixture was stirred at room temperature overnight. After completion, the solvent was removed under reduced pressure and the desired amine **10** (2.20 g, quantitative yield) was obtained as a white-yellow solid. ^{1}**H NMR** (400 MHz, MeOD) δ 1.62 (d, 3H, *J* 6.8), 1.64-1.71 (m, 2H), 1.74-1.85 (m, 4H), 1.91-2.00 (m, 2H), 4.4 (q, 1H, *J* 6.8), 4.81-4.87 (m, 1H), 6.93 (ddd, 1H, *J* 8.3, 2.5, 1.0), 6.98-6.99 (m, 2H), 7.34 (t, 1H, *J* 8.3).

Synthesis of *(R)*-3-chloro-*N*-(1-(3-(cyclopentyloxy) phenyl)ethyl)propane-1-sulfonamide 11

3-Chloropropanesulfonyl chloride (1.33 mL, 10.92 mmol) was added to the solution of amine **10** (2.20 g, 9.10 mmol) and trimethylamine (3.80 mL, 27.30 mmol) in CH_2Cl_2 at 0 °C. The reaction mixture was stirred at room temperature overnight. The reaction mixture was quenched with water (20 mL). The phases were separated and the aqueous layer was extracted with CH_2Cl_2 (10 mL). The combined organic layers were successively washed with 1N HCl (2 × 10 mL), brine (10 mL) and dried over Na_2SO_4. After evaporation of the solvent, the expected product **11** (3.10 g, 98%) was obtained as a brown oil.^{1}**H NMR** (400 MHz, $CDCl_3$) δ 1.54 (d, 3H, *J* 6.8), 1.58-1.68 (m, 2H), 1.75-1.97 (m, 6H), 2.02-2.18 (m, 2H), 2.76-2.83 (m, 1H), 2.89-2.96 (m, 1H), 3.41-3.53 (m, 2H), 4.58 (app quint, 1H, *J* 7.0, 6.8), 4.72 (d, 1H, *J* 6.8), 4.74-4.79 (m, 1H), 6.80 (dd, 1H, *J* 8.0, 2.2), 6.83 (t, 1H, *J* 2.2), 6.87 (br d, 1H, *J* 7.8), 7.23 (app t, 1H, *J* 8.0, 7.8).

Synthesis of *(R)*-3-(*N*-(1-(3-(cyclopentyloxy)phenyl) ethyl)sulfamoyl)propyl acetate 12

A suspension of chlorine **11** (3.10 g, 8.96 mmol), NaOAc (1.62 g, 19.71 mmol) and NaI (2.95 g, 19.71 mmol) in dimethylformamide was heated at 80 °C for 5h. The reaction mixture was then cooled to room temperature and stirred overnight. Water (15 mL) and EtOAc (15 mL) were added to the reaction mixture and the phases were separated. The aqueous phase was extracted with EtOAc (2 × 15 mL). The combined organic layers were washed with brine (2 × 15 mL), dried over Na_2SO_4, and then concentrated under reduced pressure. The oily residue was purified by flash-chromatography over silica gel (eluent: ^{i}Hex/EtOAc 7/3) to afford the desired ester product **12** (2.40 g, 72%) as a yellow oil.^{1}**H NMR** (400 MHz, $CDCl_3$) δ 1.54 (d, 3H, *J* 6.8), 1.58-1.68 (m, 2H), 1.76-1.86 (m, 4H), 1.88-1.98 (m, 4H), 2.00 (s, 3H), 2.65-2.72 (m, 1H), 2.79-2.86 (m, 1H), 3.93-4.04 (m, 2H), 4.58 (app quint, 1H, *J* 7.0, 6.8), 4.66 (d, 1H, *J* 7.0), 4.73-4.78 (m, 1H), 6.80 (ddd, 1H, *J* 8.0, 2.2, 0.8), 6.83 (t, 1H, *J* 2.2), 6.87 (dm, 1H, *J* 7.8), 7.26 (app t, 1H, *J* 8.0, 7.8).

Synthesis of *(R)*-*N*-(1-(3-(cyclopentyloxy)phenyl)ethyl)-3-hydroxypropane-1-sulfonamide 13

Ester **12** (2.40 g, 6.50 mmol) was dissolved in a solution of 10% HCl in methanol and the reaction mixture was heated at reflux (~80 °C) for 1h. The reaction mixture was concentrated under reduced pressure and the residue was purified by flash-chromatography over silica gel (eluent: ^{i}Hex/EtOAc 4/6) to afford the corresponding alcohol **13** (1.74 g, 82%) as a light yellow oil. ^{1}**H NMR** (400 MHz, $CDCl_3$) δ 1.54 (d, 3H, *J* 6.8), 1.58-1.68 (m, 2H), 1.75-1.97 (m, 8H), 2.76-2.83 (m, 1H), 2.88-2.95 (m, 1H), 3.56-3.66 (m, 2H), 4.59 (quint, 1H, *J* 7.0), 4.75-4.79

(m, 1H), 4.88 (d, 1H, *J* 6.8), 6.79 (dd, 1H, *J* 8.0, 2.2), 6.85 (t, 1H, *J* 2.2), 6.88 (d, 1H, *J* 7.8), 7.26 (app t, 1H, *J* 7.8, 8.0).

Synthesis of (*R*)-*N*-(1-(3-(cyclopentyloxy)phenyl)ethyl)-3-((2,4-dioxo-3,4-dihydropyrimidin-1(2H)-yl)-methoxy)propane-1-sulfonamide 2

Alcohol **13** (1.74 g, 5.31 mmol) was added to a suspension of paraformaldehyde (159.60 mg, 5.31 mmol) in trimethylsilylchloride (2.21 mL, 17.30 mmol) at room temperature. The reaction mixture was stirred until a clear solution formed. The solution was then concentrated under reduced pressure to afford the chloromethylether intermediate, which is taken to the next step without purification.

Bis(trimethylsilyl)acetamide (2.44 mL, 9.82 mmol) was added to a suspension of uracil (506 mg, 4.51 mmol) in dichloromethane (18 mL) at room temperature. The resulting mixture was stirred until a clear solution is achieved (~30-45 min). This solution was then added to the freshly prepared chloromethylether. The resulting reaction mixture was stirred at room temperature overnight. The reaction mixture was then diluted with dichloromethane (15 mL) and quenched by adding a saturated solution of sodium hydrogenocarbonate (15 mL). The aqueous phase was extracted with dichloromethane (3 × 15 mL). The combined organic extracts were dried over Na_2SO_4 and concentrated under reduced pressure. The obtained residue was purified by column chromatography (eluent: CH_2Cl_2/MeOH 97/3) to afford the desired product **2** (315 mg, 13%) as a colorless oil. **ESI-MS*:*** m/z = 452 $[M+H]^+$. **^{1}H NMR** (400 MHz, $CDCl_3$) δ 1.54 (d, 3H, *J* 6.9), 1.61-1.68 (m, 2H), 1.75-1.83 (m, 4H), 1.84-2.00 (m, 4H), 2.69-2.76 (m, 1H), 2.81-2.89 (m, 1H), 3.49-3.56 (m, 2H), 4.58 (app quint, 1H, *J* 6.8, 6.9, 7.0), 4.69 (d, 1H, *J* 6.9), 4.75-4.79 (m, 1H), 5.06 (s, 2H), 5.76 (dd, 1H, *J* 8.0, 2.2), 6.80 (ddd, 1H, *J* 8.0, 2.2, 0.8), 6.84 (t, 1H, *J* 2.2), 6.87 (d, 1H, *J* 7.6), 7.24-7.31 (m, 2H), 8.42 (br s, 1H).

Selectivity assays

For selectivity assays, the compounds to be analyzed were nano-dispensed, in dose-response curves, using an Echo 550 (Labcyte) directly in 384-well assay plates. Enzymes and substrates were diluted in their respective assay buffer and added to assay plates with Multidrop (Thermo Fisher) at a final volume of 50 µL/well (the procedures were adapted from references [5, 6]). The respective reaction mixtures were incubated for 15-30 min at RT after which 10 µL malachite green assay reagent was added to the plate. After colour development for 15 min, the absorbance of the assay plate was read at 630 nm using a Hidex Sense plate reader. The IC50 value was determined by fitting a dose response curve to the data points using nonlinear regression analysis.

The reactions were either coupled to pyrophosphatase (PPase) or phosphatase (BIP) to produce inorganic phosphate that can be detected by using the malachite green reagent. The assay conditions were for **dUTPase**: PPase coupled enzyme, 0.4 U/mL PPase, 12.5 µM dUTP, 1.18 nM dUTPase in reaction buffer containing 100 mM Tris-acetate pH 8, 40 mM NaCl, 10 mM Mg acetate, 1 mM DTT and 0.005% Tween 20, incubation 20 min; for **MTH1**: PPase coupled enzyme, 0.2 U/mL PPase, 100 µM dGTP, 4.75 nM MTH1 in reaction buffer containing 100 mM Tris-acetate pH 8, 40 mM NaCl, 10 mM Mg acetate, 1 mM DTT and 0.005% Tween 20, incubation 15 min; for **NUDT5**: BIP coupled enzyme, 10 U/mL BIP, 50 µM ADPR, 6 nM NUDT5 in reaction buffer containing 100 mM Tris-acetate pH 8, 40 mM NaCl, 10 mM Mg acetate, 1 mM DTT and 0.005% Tween 20, incubation 15 min; for **NUDT12**: BIP coupled enzyme, 10 U/mL BIP, 50 µM Beta-NADH, 20 nM NUDT12 in reaction buffer containing 100 mM Tris-acetate pH 8, 40 mM NaCl, 10 mM Mg acetate, 1 mM DTT and 0.005% Tween 20, incubation 30 min; for **NUDT15**: PPase coupled enzyme, 0.2 U/mL PPase, 100 µM dGTP, 8 nM NUDT15 in reaction buffer containing 100 mM Tris-acetate pH 8, 40 mM NaCl, 10 mM Mg acetate, 1 mM DTT and 0.005% Tween 20, incubation 15 min; for **dCTPase** (His tag): PPase coupled enzyme, 0.2 U/mL PPase, 35 µM dCTP and 35 nM dCTPase in a reaction buffer containing 100 mM Tris-acetate pH 8, 100 mM KCl, 10 mM Mg acetate, 1 mM DTT and 0.005% Tween 20, incubation 40 min; for **ITPA**: PPase coupled enzyme, 0.2 U/mL PPase, 25 µM ITP, 0.1 nM ITPA in reaction buffer containing 100 mM Tris-acetate pH 8, 50 mM Mg acetate, 1 mM DTT and 0.005% Tween 20, incubation 20 min; for **SAMHD1**: PPase coupled enzyme, 12.5 U/mL PPase, 25 µM dGTP, 350 nM SAMHD1 in reaction buffer containing 25 mM Tris-acetate pH 8, 40 mM NaCl, 1 mM $MgCl_2$, 1 mM TCEP and 0.005% Tween 20, incubation 20 min followed by addition of 20 µL a solution of of EDTA at 7.9 mM prior to addition of the malachite green reagent.

Computational docking of compounds 1 and 2 into human dUTPase

All calculations were performed using Schrodinger Suite 2016-1 (Schrödinger) running on a Linux workstation. 3ARA.pdb was prepared using the Protein Preparation Wizard [7]. The structure was pre-processed by assigning bond orders, adding hydrogens, creating zero-order bonds to metals, creating disulfide bridges, and deleting waters beyond 5.0 Å from heteroatomic (het) groups. Of the two Mg atoms present in the structure only the one bound in the central pore of the homotrimer was preserved. Protonation and metal charge states were then generated for the het groups at pH 7.0. The states with the lowest penalties were selected for the next steps. The states of hydroxyl,

Asn, Gln and His residues were automatically optimized using ProtAssign, and water orientations were sampled. All waters with less than 3 H-bonds to non-waters were removed, which only left the conserved waters bridging the ligand uracil moieties with Gly97 and Val112 in each of the 3 active sites. Finally, a minimization was performed with heavy atoms restrained and hydrogens unrestrained until a convergence for the heavy atoms reached an RMSD of 0.30 Å, using the OPLS2005 force field [8]. Glide [9] docking grids were generated by centering the enclosing box on the centroid of the ligand MKH belonging to chain A. The size of the enclosing box was set to cover ligands similar in size to MKH. No constraints, rotatable groups or excluded volumes were defined. Compound **1** and compound **2** were built manually in the Maestro GUI, ensuring the correct stereochemistry on their chiral centers (*S* for compound **1** and *R* for compound **2**). LigPrep was then used to generate low-energy 3D starting conformations for docking. OPLS2005 was chosen as the force field for minimization. Possible ionization and tautomeric states were generated using Epik [10] at pH 7.0 ± 2.0. Chiralities were determined from the 3D starting structures. The prepared ligands were then docked using Glide SP [9]. OPLS2005 was chosen as the force field. Flexible ligand sampling was applied, allowing inversion of nitrogens and sampling of ring conformations. Nonplanar amide conformations were penalized. Epik state penalties were added to the docking scores. Up to 5 poses per ligand were saved. The Pose Viewer was used to analyze the docking results.

Phosphorylated H2A.X analysis

Cells, treated as described for the resazurin survival assay, were fixed with 4% paraformaldehyde for 20 minutes. After permeabilization with PBS containing 0.5% TritonX-100, samples were blocked with 3% BSA in PBS and incubated with primary anti-phospho-Histone H2A.X (Ser139) antibody (γH2A.X; Millipore) overnight at 4 °C. Secondary goat anti-rabbit IgG antibody conjugated with Alexa Fluor® 488 was subsequently added for one hour incubation. DNA was counterstained using DAPI. Immunostainings were imaged using the 10x objective on the ImageXpress and analyzed with the CellProfiler 2.1.1. software. Mean intensities of Alexa Fluor® 488 were assessed from 9 sites per well.

REFERENCES

1. Baykov AA, Evtushenko OA, Avaeva SM. A malachite green procedure for orthophosphate determination and its use in alkaline phosphatase-based enzyme immunoassay. Analytical biochemistry. 1988; 171:266-270.
2. Nguyen C, Ruda GF, Schipani A, Kasinathan G, Leal I, Musso-Buendia A, Kaiser M, Brun R, Ruiz-Perez LM, Sahlberg BL, Johansson NG, Gonzalez-Pacanowska D, Gilbert IH. Acyclic nucleoside analogues as inhibitors of Plasmodium falciparum dUTPase. Journal of Medicinal Chemistry. 2006; 49:4183-4195.
3. Keuser C, Pindur U. Oligopyrrole carboxamides linked with a nucleobase as potential DNA minor groove binding ligands: synthesis, DNA binding and biological evaluation. Pharmazie. 2006; 61:261-268.
4. Fukuoka M, Yokogawa T, Miyahara S, Miyakoshi H, Yano W, Taguchi J, Takao Y. Taiho Pharmaceutical Co., Ltd. Novel uracil compound or salt thereof having human deoxyuridine triphosphatase inhibitory activity. United States Patent US 2011/0082163. 2011 Apr 7.
5. Herold N, Rudd SG, Ljungblad L, Sanjiv K, Myrberg IH, Paulin CB, Heshmati Y, Hagenkort A, Kutzner J, Page BD, Calderon-Montano JM, Loseva O, Jemth AS, et al. Targeting SAMHD1 with the Vpx protein to improve cytarabine therapy for hematological malignancies. Nat Med. 2017; 23:256-263.
6. Gad H, Koolmeister T, Jemth AS, Eshtad S, Jacques SA, Strom CE, Svensson LM, Schultz N, Lundback T, Einarsdottir BO, Saleh A, Gokturk C, Baranczewski P, et al. MTH1 inhibition eradicates cancer by preventing sanitation of the dNTP pool. Nature. 2014; 508:215-221.
7. Sastry GM, Adzhigirey M, Day T, Annabhimoju R, Sherman W. Protein and ligand preparation: parameters, protocols, and influence on virtual screening enrichments. J Comput Aided Mol Des. 2013; 27:221-234.
8. Shivakumar D, Williams J, Wu Y, Damm W, Shelley J, Sherman W. Prediction of Absolute Solvation Free Energies using Molecular Dynamics Free Energy Perturbation and the OPLS Force Field. J Chem Theory Comput. 2010; 6:1509-1519.
9. Friesner RA, Banks JL, Murphy RB, Halgren TA, Klicic JJ, Mainz DT, Repasky MP, Knoll EH, Shelley M, Perry JK, Shaw DE, Francis P, Shenkin PS. Glide: a new approach for rapid, accurate docking and scoring. 1. Method and assessment of docking accuracy. J Med Chem. 2004; 47:1739-1749.
10. Greenwood JR, Calkins D, Sullivan AP, Shelley JC. Towards the comprehensive, rapid, and accurate prediction of the favorable tautomeric states of drug-like molecules in aqueous solution. J Comput Aided Mol Des. 2010; 24:591-604.

SUPPLEMENTARY FIGURES

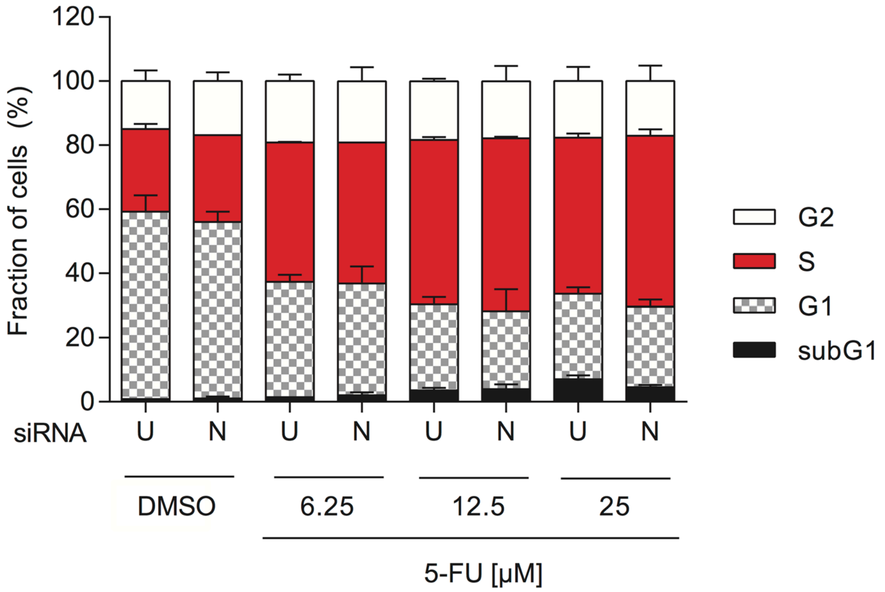

Supplementary Figure 1: FACS analysis showing 5-FU-induced cell cycle alterations in untransfected (U) and siNon-t (N) transfected cells, included as control for the experiment shown in Figure 1C-D. Cells were transfected with siNon-t or not transfected for 48 hours, re-seeded and, 24 hours later, treated with the indicated concentrations of 5-FU for another 48 hours. DNA content was stained with PI and analyzed by FACS. Data shown as average ± SEM from two independent experiments.

Compound 1

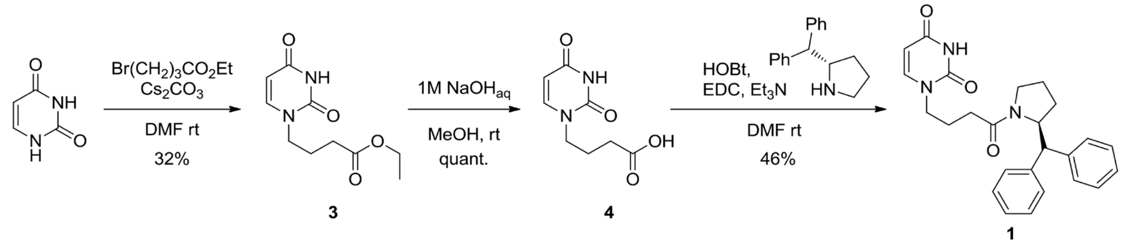

Compound 2

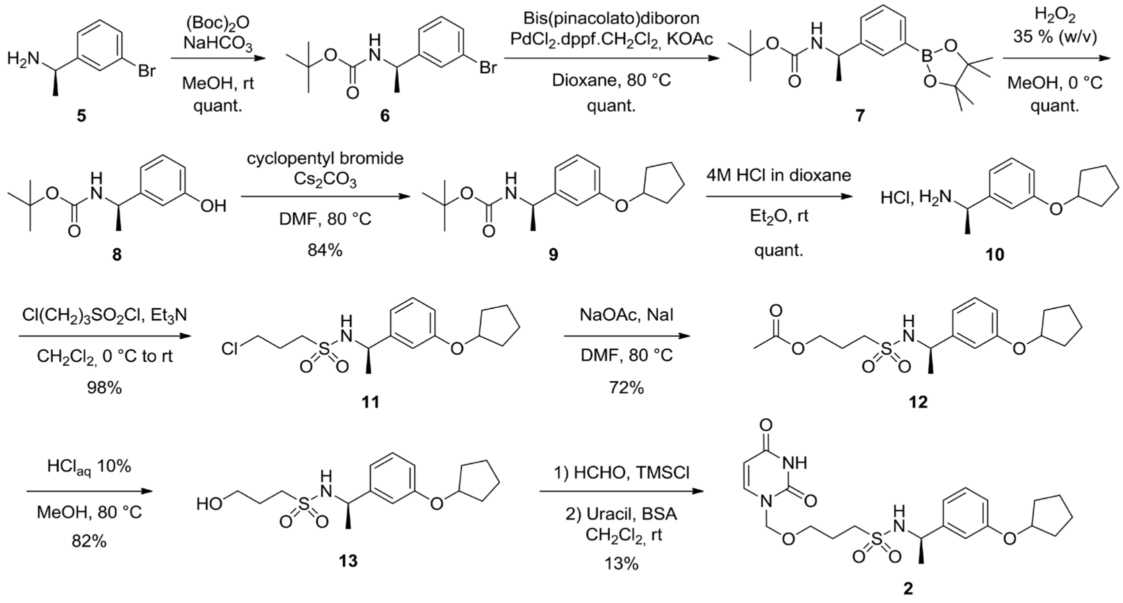

Supplementary Figure 2: Synthetic routes for the dUTPase inhibitors 1 and 2. A detailed description can be found in the Supplementary Materials and Methods. Abbreviations: quantitative: quant., room temperature: rt.

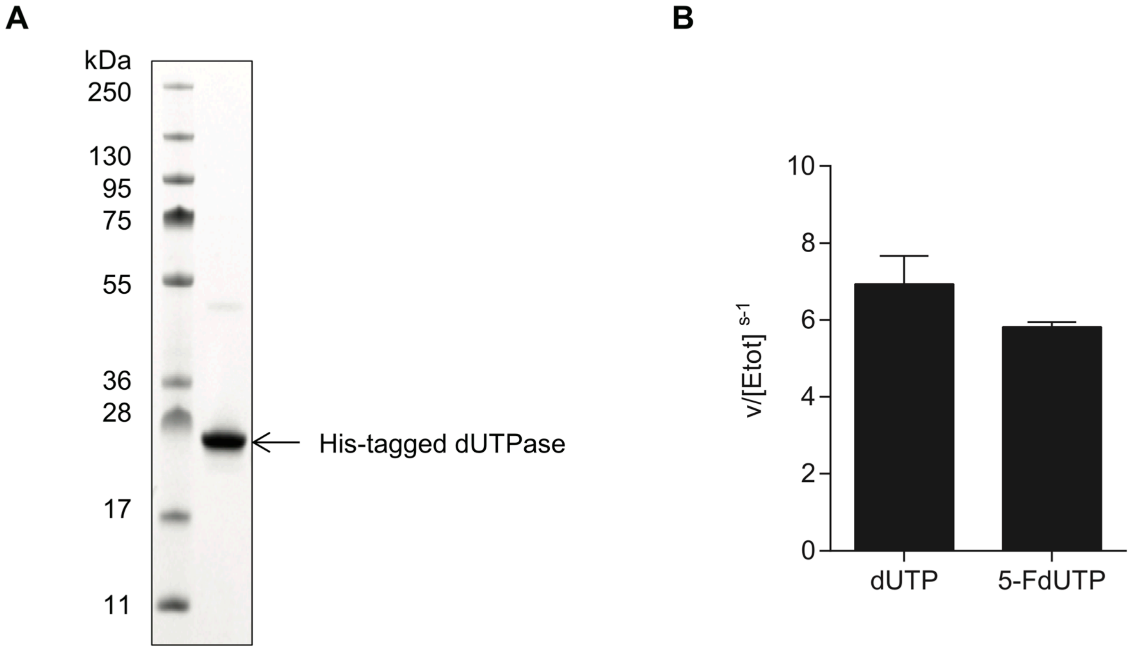

Supplementary Figure 3: (A) Purified His-tagged dUTPase protein used for *in vitro* studies. Human dUTPase was expressed and purified from bacterial lysates, separated by SDS-PAGE and stained with Coomassie to assess the purity of the protein. **(B)** dUTPase-catalyzed hydrolysis of dUTP and 5-FdUTP assessed by malachite green assay. Data shown as average ± SEM from three independent experiments measured in duplicate. The data is presented as v (hydrolyzed substrate [µM] per second) per total enzyme concentration [µM].

	Compound **1**	Compound **2**
MTH1	> 100	> 100
NUDT5	> 100	> 100
NUDT12	> 100	> 100
NUDT15	> 100	> 100
dCTPase	> 100	> 100
ITPase	> 100	> 100
SAMHD1	> 100	> 100

Supplementary Figure 4: Selectivity profile of compounds 1 and 2 towards NUDIX enzymes (MTH1, NUDT5, NUDT12, NUDT15) and other proteins with known nucleoside triphosphate pyrophosphatase (dCTPase, ITPase) or phosphohydrolase (SAMHD1) activity. IC_{50} expressed as µM values.

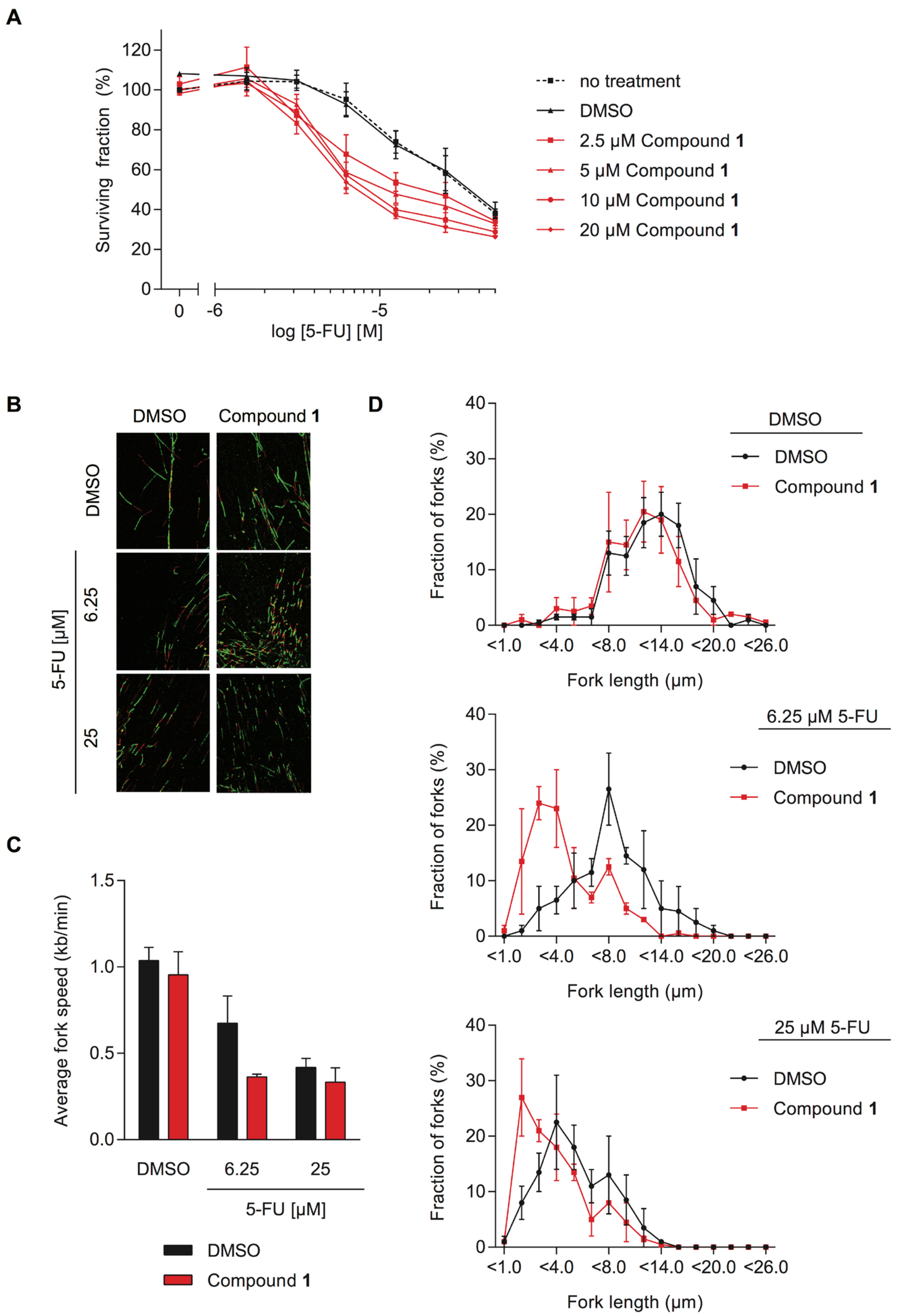

Supplementary Figure 5: (A) Resazurin experiment assessing the viability of SW620 cells co-treated with 5-FU and compound **1** at the indicated concentrations for 72 hours. Values were normalized against the untreated control (-). Data shown as average ± SEM from three independent experiments performed in duplicate. **(B)** Representative images of DNA replication forks from cells treated with the indicated concentration of 5-FU in combination with respectively 10 μM of compound **1** or a DMSO control for 48 hours. **(C)** Average fork speed during IdU labeling of cells treated as described in **Supplementary Figure 5B**. Data shown as average ± SEM from two independent experiments. **(D)** Distribution of IdU labeled fiber length of the experiment shown in **Supplementary Figure 5B**. Data shown as average ± SEM from two independent experiments.

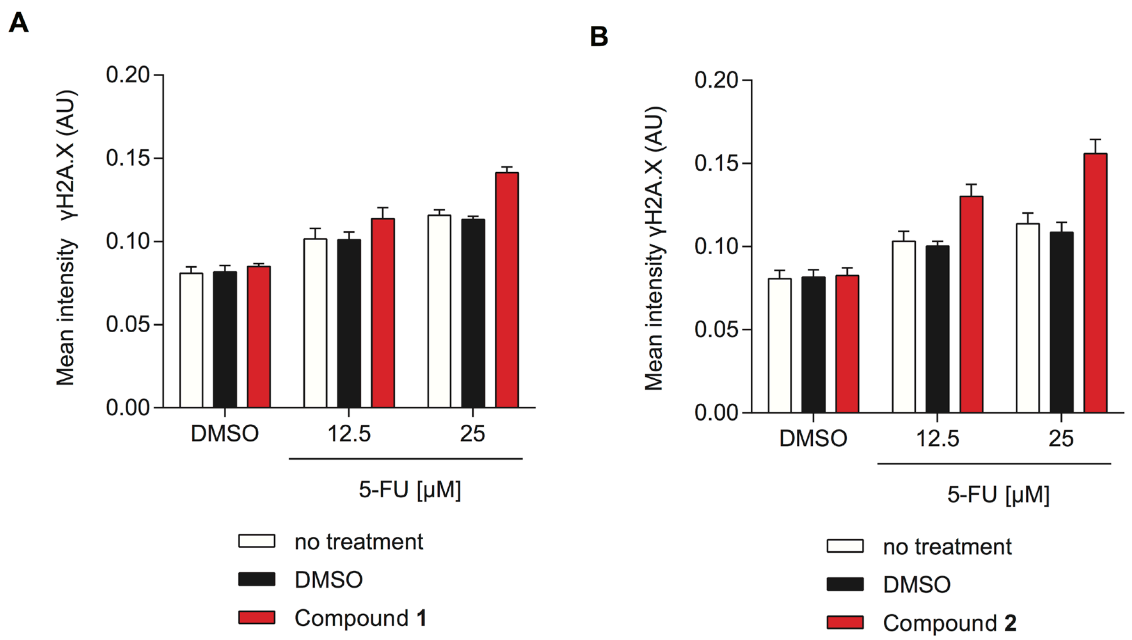

Supplementary Figure 6: Average intensity of H2A.X phosphorylation in cells treated for 72 hours with 5-FU in combination with 2.5 μM of compound 1 (A) or 2 (B) or a DMSO control, measured by automated microscopy. Data shown as average ± SEM from three independent experiments. Abbreviation, AU: arbitrary unit.

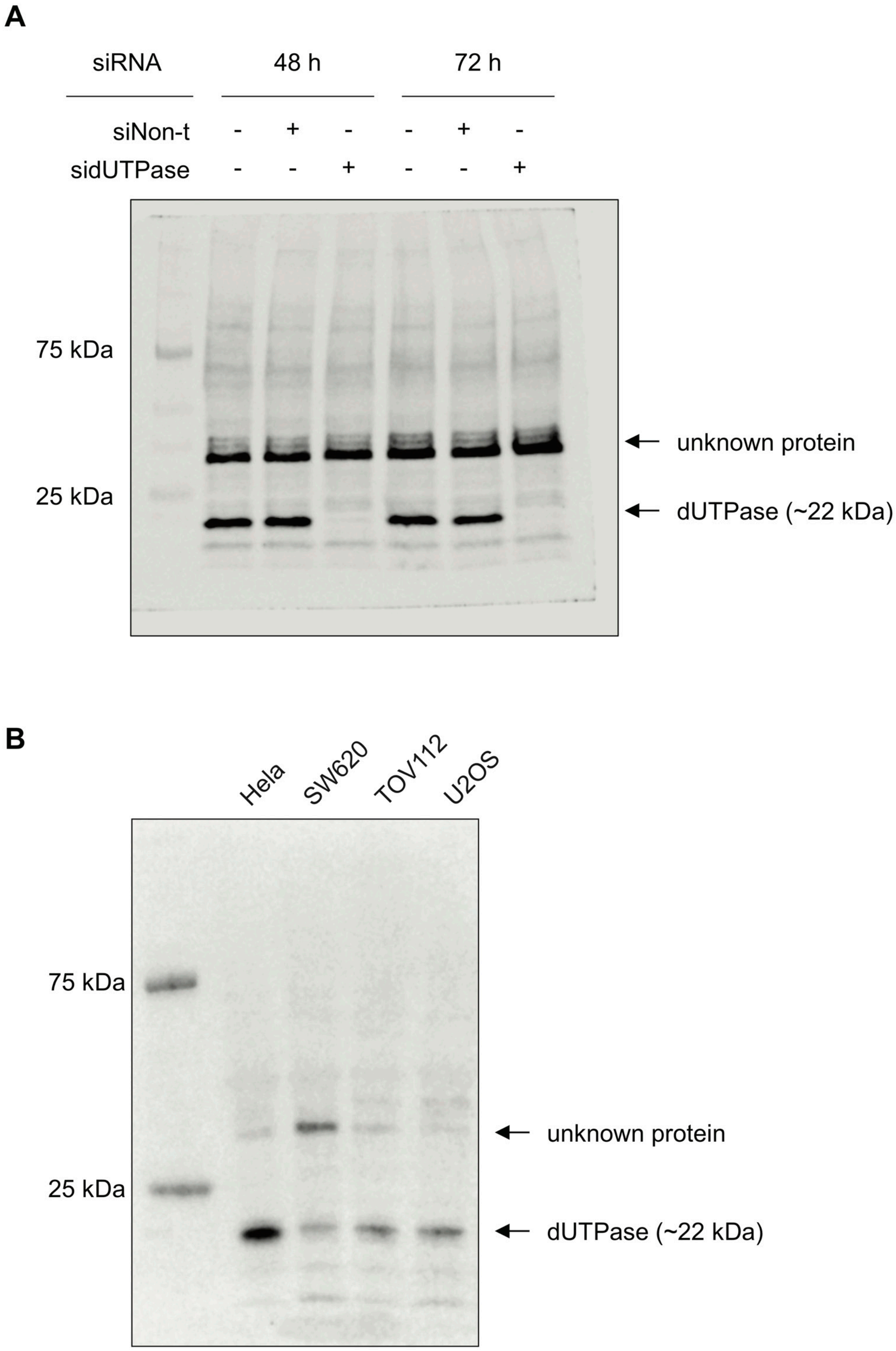

Supplementary Figure 7: (A) Image of the uncut membrane of the Western Blot experiment shown in **Figure 1A**. The membrane was stained with the rat anti-dUTPase antibody to depict specificity of the antibody. dUTPase knockdown resulted in depletion of a band around 22 kDa, the height of expected dUTPase (mitochondrial variant 23 kDa and nuclear dUTPase 22 kDa), indicating that this band contains dUTPase. A second band was observed around 35 kDa, which could not be depleted by siRNA treatment, suggesting that the antibody binds an unknown protein. **(B)** Image of the uncut membrane of the Western Blot shown in **Figure 5D**. The ladder Precision Plus Protein™ Dual Color Standards from Bio Rad was used.
